# UnTWISTing intestinal fibrosis: single-cell transcriptomics deciphers fibroblast heterogeneity, uncovers molecular pathways, and identifies therapeutic targets

**DOI:** 10.1172/JCI184112

**Published:** 2024-09-17

**Authors:** Giovanni Santacroce, Antonio Di Sabatino

**Affiliations:** 1 Department of Internal Medicine and Medical Therapeutics, University of Pavia, Pavia, Italy.; 2 First Department of Internal Medicine, San Matteo Hospital Foundation, Pavia, Italy.

## Abstract

Intestinal fibrosis is a severe complication of Crohn’s disease, often requiring surgical intervention. Despite extensive research efforts, an effective treatment to prevent or reverse intestinal fibrosis remains elusive. In this issue of the *JCI*, Zhang, Wang, and colleagues employed single-cell RNA sequencing to uncover mechanisms of the fibrotic process. They identified a key fibroblast subset of TWIST1^+^FAP^+^ cells that interacts with CXCL9^+^ macrophages. TWIST1 emerged as a central regulator of the fibrotic microenvironment, representing a promising therapeutic target for effectively treating intestinal fibrosis.

## Intestinal fibrosis and single-cell transcriptomics

Intestinal fibrosis is a common complication of Crohn’s disease (CD), and it leads to strictures and obstructive symptoms (1). It affects more than 50% of patients with CD and often requires surgery. Over the past decades, substantial scientific efforts have been made to untangle the puzzle of intestinal fibrosis (2). The core of the fibrogenesis process is represented by inflammation-triggered myofibroblast production of extracellular matrix (ECM), driven by TGF-β stimulation and mediated by stromal-immune cell interactions (2). Additionally, the transition of epithelial and endothelial cells into activated myofibroblasts (3), the smooth muscle cell hyperplasia/hypertrophy (4), and the creeping fat (5) all contribute to stricture formation. Despite advances in our understanding, a medication capable of reversing or treating fibrosis is still lacking, representing a pressing unmet need in the field of inflammatory bowel disease (6).

The main limitation in advancing this field is the focus on surface-level elements of the process, overlooking the hidden and complex aspects of intestinal fibrosis, which involve cellular heterogeneity, cell-cell crosstalk, molecular interactions, and signaling pathways. The recent application of omics techniques in intestinal fibrosis research, including single-cell RNA sequencing (scRNA-Seq), has shown promise in detailed mapping of tissue architecture and in-depth characterization of cellular and molecular mechanisms at a single-cell level (7). These advancements can broaden our understanding, helping identify novel therapeutic targets and potentially solving intestinal fibrosis.

## Fibroblast heterogeneity

The work by Zhang, Wang, and colleagues in this issue of the *JCI* represents a notable illustration of the transformative potential of scRNA-Seq in intestinal fibrosis research (8). By applying this technique to fibrotic and nonfibrotic ileal samples from patients with CD, the authors identified distinct subsets of fibroblasts, including NT5E^+^ fibroblasts, FAP^+^ fibroblasts, CCL11^+^ fibroblasts, and FDFR2^+^ fibroblasts. Of interest, FAP^+^ fibroblasts, originating from inflammatory FGFR2^+^ fibroblasts, emerged as the predominant subset increased within fibrotic areas and crucially involved in ECM formation. This study corroborates and expands upon previous ex vivo observations of FAP upregulation in stenotic CD mucosa (9), suggesting a potential role for FAP inhibition in restoring ECM homeostasis and mitigating fibrosis. Other studies have comprehensively characterized single-cell populations in CD strictures, revealing distinct fibroblast subpopulations specific to the mucosa and submucosa, such as the CXCL14^+^ and MMP/WNT5A^+^ fibroblasts, and to the creeping fat, including GREM1^+^ and RFLNB^+^ fibroblasts (10, 11). Together, these omics-enabled investigations have the potential to unravel the cellular heterogeneity of fibrotic strictures, pinpointing the subsets of cells pivotal to the disease process and highlighting potential cellular therapeutic targets for further exploration.

## Cell-cell interaction

scRNA-Seq enables the characterization of cellular subpopulations involved in intestinal fibrosis and can help also in elucidating the cell-cell interactions driving the fibrotic process. Accumulating data highlight the pivotal role of stromal-immune crosstalk in fibrosis pathogenesis (12). Stromal cells support innate and adaptive immune cells, recruiting neutrophils, monocytes, and T cells to regulate chronic inflammation. Simultaneously, activated immune cells, such as M2-polarized macrophages, contribute to fibrosis by stimulating fibroblast ECM production (13). Notably, the scRNA-Seq analysis performed by Zhang et al. identified distinct macrophage clusters, with CXCL9^+^ macrophages, involved in chemotaxis and ECM organization, which were enriched in fibrotic areas (8). These CXCL9^+^ macrophages correlated with FAP^+^ fibroblasts, colocalizing within fibrotic sites and displaying interaction patterns involving IL-1β and TGF-β pathways, as assessed by NicheNet analysis (14). A broader interaction analysis performed by Mukherjee et al. identified major populations with strong incoming receptor signals within the lamina propria of CD strictures, including myeloid cells (CXCL9/CXCL10^hi^, inflammatory monocytes, NLRP3^hi^, and Met-allothionein^hi^), T/NK cells, endothelial cells, MMP/WNT5A^+^ fibroblasts, and pericytes (10). Additionally, Mukherjee and colleagues identified lamina propria fibroblasts, including CXCL14/ADAMDEC1^+^, MMP/WNT5A^+^, CXCL14/F3/PDGFRA^+^, and ECM^hi^ cells, as having the major signal-sending role, highlighting their critical role in the fibrotic interaction hub (10). Enhanced comprehension of diverse cell subsets implicated in intestinal fibrosis and their interactions holds promise for identifying novel therapeutic targets. Targeting multiple cells, particularly the ligands and receptors governing their interactions, may be crucial for effectively treating fibrosis in the future.

## Molecular pathways

Going further, scRNA-Seq offers an incredible opportunity to delve into the intricate mechanisms of intestinal fibrosis, uncovering the molecular pathways involved. Using single-cell regulatory network inference and clustering (SCENIC) analysis, Zhang, Wang, and colleagues identified TWIST1 as a critical factor regulating the differentiation of FAP^+^ fibroblasts and their interaction with CXCL9^+^ macrophages, orchestrating fibrosis (8). This finding was confirmed ex vivo in primary human intestinal fibroblasts and in vivo in mouse models.

TWIST1 is a basic helix-loop-helix transcription factor controlled by several upstream regulators, including STAT3, HIF, NF-κB, the Wnt/β-catenin pathway, and the TGF-β/Smad pathway. (15) TWIST1 promotes fibrotic disorders via several profibrotic mechanisms. Recent human multi-omic single-cell analyses have demonstrated the ability of TWIST1 to regulate myofibroblast activation in the lung (16). Furthermore, TWIST1 drives renal fibrosis by regulating M2 polarization through galectin-3 and inhibiting fatty acid oxidation (17, 18). Additionally, TWIST1 plays a role in modulating epithelial-mesenchymal and endothelial-mesenchymal transitions in lung and kidney fibrosis (19, 20).

It is plausible that TWIST1 serves as a central regulator also in intestinal fibrosis. Zhang et al. have shed the light on an important role for TWIST1 in this context (8). TWIST1, by potentially regulating several cells and mechanisms associated with intestinal fibrosis, represents an extremely promising therapeutic target. Indeed, the degradation of TWIST1 through its inhibitor harmine has shown promise in suppressing fibroblast activation and ECM production both ex vivo and in transgenic TWIST1-knockout mouse models (8). Although further studies are required to elucidate and confirm TWIST1 as a therapeutic target in CD-related fibrosis, its identification demonstrates the potential of scRNA-Seq in uncovering therapeutic targets.

## Conclusion

Overall, the study by Zhang, Wang, and colleagues confirms that scRNA-Seq is a formidable tool in our arsenal to unravel the complexities of CD-related intestinal fibrosis (8). This advanced technique allows for an in-depth characterization of critical cells involved in the pathogenic process, the key interactions within the fibrotic microenvironment, and the molecular pathways that regulate it. These insights are crucial for identifying novel effective therapeutic targets and facilitating the development of agents capable of reversing and treating fibrosis.

However, transitioning these advanced techniques from bench to bedside is hindered by several challenges, including the need for procedure standardization, proper patient population selection, and large sample sizes needed to identify robust patterns accounting for high interpatient variability and study heterogeneity. Additionally, the high cost and expertise required pose substantial limitations. The vast amount of data generated by these techniques can be overwhelming and difficult to interpret, underscoring the need for machine-learning models to standardize the process, deconvolute data, and highlight critical information for researchers and physicians (21).

**Figure 1:**
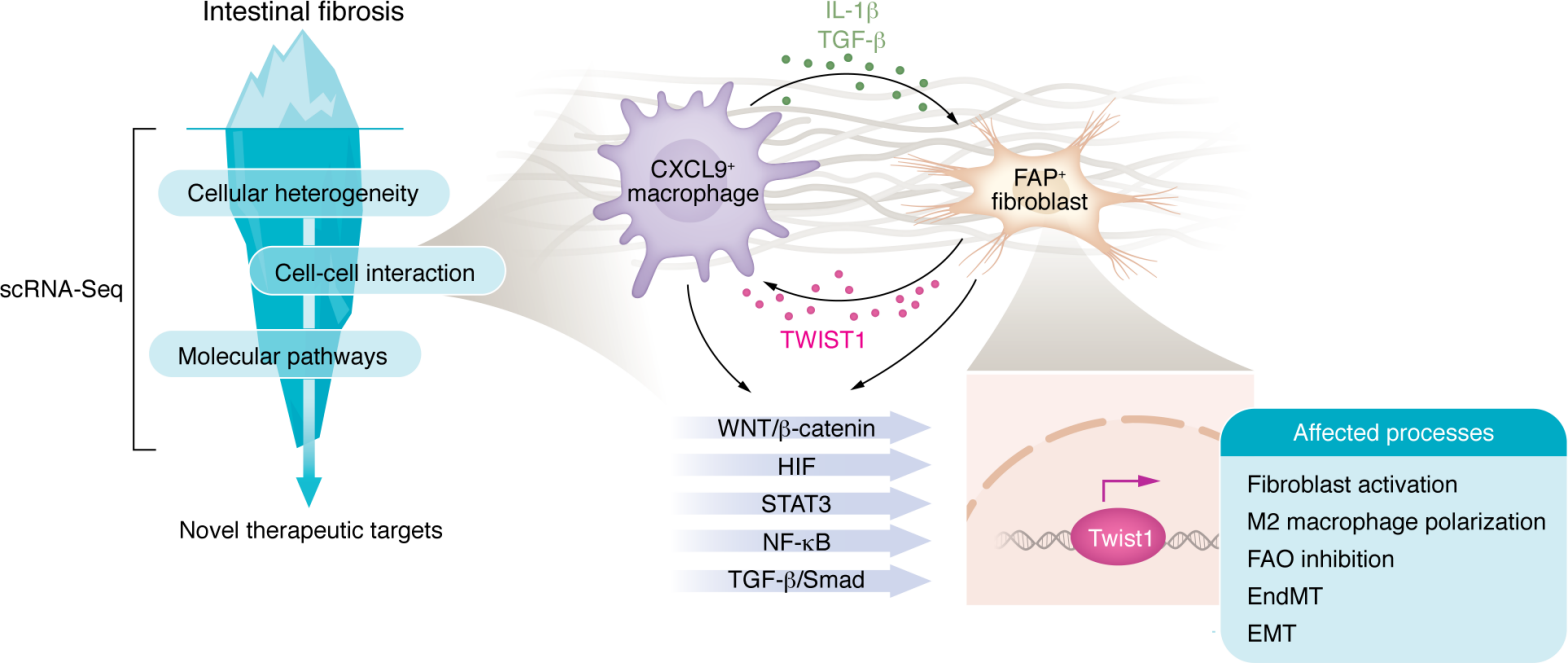
Single-cell RNA sequencing unveils the hidden iceberg in Crohn’s disease–related intestinal fibrosis. scRNA-Seq enables in-depth characterization of cellular heterogeneity within intestinal strictures, key interactions in the fibrotic microenvironment, and the molecular pathways that regulate it, ultimately facilitating the identification of therapeutic targets. In this issue of the *JCI*, Zhang, Wang, and colleagues used scRNA-Seq to discover a critical fibroblast subset of FAP^+^ cells interacting with CXCL9^+^ macrophages through the IL-1β and the TGF-β pathways (8). The transcription factor TWIST1, which is regulated by multiple upstream factors, acts as a central regulator of the intestinal fibrotic microenvironment. TWIST1 has the potential to modulate fibroblast activation, M2 macrophage polarization, epithelial-mesenchymal transition (EMT), endothelial-mesenchymal transition (EndMT), and fatty acid oxidation (FAO), making it an intriguing therapeutic target for further exploration.

Despite these challenges, the results obtained so far are encouraging. They have preliminarily shown the ability of scRNA-Seq to uncover the hidden underwater portion of the fibrosis iceberg (Figure 1), holding promise for ultimately striking down intestinal fibrosis.

## Version 1 09/17/2024

Electronic publication
